# Structural Microangiopathies in Skeletal Muscle Related to Systemic Vascular Pathologies in Humans

**DOI:** 10.3389/fphys.2020.00028

**Published:** 2020-02-05

**Authors:** Oliver Baum, Jonathan Bernd, Samuel Becker, Adolfo Odriozola, Benoît Zuber, Stefan A. Tschanz, Andreas Zakrzewicz, Stuart Egginton, Janine Berkholz

**Affiliations:** ^1^Institut für Physiologie, Charité – Universitätsmedizin Berlin, Berlin, Germany; ^2^Institute of Anatomy, University of Bern, Bern, Switzerland; ^3^School of Biomedical Sciences, Faculty of Biological Sciences, University of Leeds, Leeds, United Kingdom

**Keywords:** capillaries, human pathologies, morphometry, skeletal muscle, transmission electron microscopy

## Abstract

It is unclear how microangiopathic changes in skeletal muscle vary among systemic vascular pathologies. We therefore analyzed the capillary fine structure in skeletal muscle from patients with arterial hypertension (HYPT), diabetes mellitus type 2 (T2DM) or intermittent claudication – peripheral arterial disease (IC/PAD). Tablet-based image analysis (TBIA) was carried out to largely re-evaluate 5,000 transmission electron micrographs of capillaries from 126 vastus lateralis biopsies of 75 individuals (HYPT, T2DM or IC/PAD patients as well as healthy individuals before and after endurance exercise training) used in previous morphometric studies, but assessed using stereological counting grids of different sizes. Serial block-face scanning electron microscopy (SBFSEM) of mouse skeletal muscle was used for validation of the particular fine structural events observed in human biopsies. The peri-capillary basement membrane (BM) was 38.5 and 45.5% thicker (*P* < 0.05) in T2DM and IC/PAD patients than in the other groups. A 17.7–39.6% lower (*P* < 0.05) index for intraluminal endothelial cell (EC) surface enlargement by projections was exclusively found in T2DM patients by TBIA morphometry. The proportion of capillaries with disrupted BM between pericytes (PC) and EC was higher (*P* < 0.05) in HYPT (33.2%) and T2DM (38.7%) patients than in the control group. Empty EC-sockets were more frequent (*P* < 0.05) in the three patient groups (20.6% in HYPT, 27.1% in T2DM, 30.0% in IC/PAD) than in the healthy individuals. SBFSEM confirmed that EC-sockets may exhibit close proximity to the capillary lumen. Our comparative morphometric analysis demonstrated that structural arrangement of skeletal muscle capillaries is more affected in T2DM than in HYPT or IC/PAD, although some similar elements of remodeling were found. The increased frequency of empty EC-sockets in the three patient groups indicates that the PC-EC interaction is commonly disturbed in these systemic vascular pathologies.

## Introduction

Capillaries are the major sites of diffusive oxygen exchange and removal of catabolic products in all tissue, and thus represent a crucial structural element defining functional capacity of the cardiovascular system. Pathological changes of the capillary bed in structure and function (“microangiopathies”) have been correlated with various diseases and morbidities (O’Riely, 2015), e.g., in many patients with diabetes mellitus (DM) these are most evident in the kidney (diabetic nephropathy, DN) and the eye (diabetic retinopathy, DR). During the initial clinically silent period of DN, the glomerular basement membrane (BM) expands and podocytes undergo apoptosis (Kanwar et al., 2011; Vallon and Komers, 2011). As a result, a transient hyperfiltration through the glomerular capillary loops is established, which together with glomerular hypertony and hyperperfusion (due to constriction of efferent arterioles) progresses to clinically manifested micro- and then macroalbuminuria, and finally kidney necrosis (Kanwar et al., 2011; Vallon and Komers, 2011). DR basically affects the retinal capillary bilayer and usually begins with degeneration of pericytes (PC), BM thickening, development of microaneurysms, formation of shunt vessels and multiple capillary occlusions (Kur et al., 2012). The vessel walls become leaky due to endothelial cell (EC) apoptosis, inducing capillaries to undergo rarefaction (Kur et al., 2012). These processes combine to establish retinal ischemia, which elicits VEGF-mediated angiogenesis as a compensatory response, and excessive capillary proliferation then leads to visual disturbances or blindness (Kur et al., 2012). Interestingly, while the pathophysiological effects are not as devastating as in DN or DR, diabetic microangiopathy is also manifested in skeletal muscle, e.g., the peri-capillary BM (CBM) is thickened in type-2 diabetes mellitus (T2DM) patients (Williamson and Kilo, 1983; Mortensen et al., 2019). Furthermore, the turnover of PC is accelerated (Tilton et al., 1981), and EC apoptosis rates are increased (Vracko and Benditt, 1970).

As some modifications in capillary fine structure (e.g., CBM thickening and PC degeneration) develop consistently in many tissues of DM patients, it has been speculated that these changes represent general hallmarks for diabetic microangiopathy (Tsilibary, 2003). In contrast, other functional and structural changes in the microcirculation (e.g., EC apoptosis and angiogenesis) appear to occur only in specific organs, depending upon tissue architecture and topology of the capillary bed. Interestingly, other reports (Makitie, 1977; Hernandez et al., 1999) indicate that the capillary phenotype in skeletal muscle is not only altered in DM patients but also in patients with other chronic systemic vascular pathologies, such as arterial hypertension (HYPT) and peripheral arterial disease (PAD). Thus, it remains unclear whether remodeling of the capillary phenotype manifested in DM is specific for diabetic microangiopathy, or reflective of a more generic repertoire in pathology.

The fine structure of capillaries may be described quantitatively by morphometry using a suitable analysis system (e.g., a stereological counting grid) on electron micrographs with transversely sectioned capillary profiles (Weibel, 1979; Egginton et al., 1996). However, the dimensions of the test system has a significant influence on the outcome of a morphometric analysis, e.g., the periodic spacing (pitch) of a counting grid influences the sensitivity of counting points and crossing intersections to the grid orientation relative to the superimposed structure of interest. While geometric probability theory dictates that adequate replication may ameliorate such effects for average tissue composition, analysis of individual profile characteristics critically depends on analytical resolution. Accordingly, reproducibility of morphometric analyses tends to deviate between studies using counting grids with wide periodic spacing (Weibel, 1979; Egginton et al., 1996).

To overcome the problem of different analytical scales, and avoid any assumption associated with model-based analysis, we developed tablet-based image analysis (TBIA): a morphometric method with a periodic spacing of 1 pixel (Bigler et al., 2016). This may be particularly useful when analyzing objects of low frequency, as rarely observed structures can be very informative but details may not be adequately captured by the point counting or line intercept approach. For example, the mean BM thickness of 15 randomly selected capillaries obtained by TBIA was within the reference range defined by two established measurement techniques (Siperstein et al., 1973; Williamson et al., 1976), whereas the corresponding values obtained with counting grids of different test line spacing deviated by up to 100% from reference values (Bigler et al., 2016). In addition, TBIA was applied almost three times faster and generated more reproducible data than grid morphometry. On the other hand, TBIA was more prone to obliquely sectioned capillary profiles than grid morphometry as a result of greater resolution. However, this drawback of TBIA can be avoided if only capillary profiles with an aspect ratio of less than 1.2 are included in the analysis (Siperstein et al., 1973; Williamson et al., 1976).

We have previously quantified the fine structure of capillaries in skeletal muscles of patients with HYPT (Gliemann et al., 2015), T2DM (Mortensen et al., 2019), and PAD (Baum et al., 2016). Following standard practice these morphometric studies were conducted using counting grids, but unfortunately with different periodic spacing. Thus, although the study design allowed internal evaluation, cross-study comparisons were not possible to adequately assess whether there are significant differences in capillary structure among these patient groups. In the present investigation we applied TBIA to largely re-evaluate 5,000 electron micrographs showing capillary profiles from our previous studies (Baum et al., 2015, 2016; Gliemann et al., 2015). Since all biopsies originated from the same muscle and were subjected to the same sample preparation before morphometry (chemical fixation, sectioning, and sampling), we were able to quantitatively compare differences in capillary fine structure across groups. Our comparative morphometric analysis revealed that several compartments and subcompartments of capillaries in human skeletal muscle undergo specific, as well as common structural alterations in HYPT, T2DM and PAD patients. We therefore recommend to avoid the term “diabetic microangiopathy” for modifications in capillary phenotype observed in skeletal muscle, and to use instead the more general term “structural microangiopathy.”

## Materials and Methods

### Study Participants and Muscle Biopsies

Biopsies of the vastus lateralis muscle (VL) were originally collected from participants in five studies conducted at the Department of Anatomy, University of Bern (Rosler et al., 1985; Suter et al., 1995), the University of Copenhagen (Nyberg et al., 2012; Winding et al., 2018), or the University of the Sunshine Coast, Australia (Walker et al., 2016), as described in detail in Table 1 and Supplementary Table 1. Anthropometric characteristics of the study participants were provided in the original publications (above) and are summarized as follows: Study 1: healthy subjects (*n* = 10), muscle biopsies were taken before and after 8 weeks endurance exercise; Study 2: healthy subjects (*n* = 12), muscle biopsies were taken before and after 6 months mild endurance exercise, six participants underwent angiogenesis (angiogenesis-responder, AR), while other six participants lacked an angiogenic response (non-angiogenesis-responder; NR); Study 3: arterial hypertension (HYPT) patients (*n* = 10) and age-matched healthy controls (*n* = 9), muscle biopsies were taken before and after 8 weeks endurance exercise; Study 4: T2DM patients (*n* = 10), muscle biopsies were taken before and after 8 weeks endurance exercise; Study 5: intermittent claudication/early stage peripheral arterial disease (IC/PAD) patients (*n* = 14) and healthy controls (*n* = 10). Three of the IC/PAD patients simultaneously suffered from T2DM and 11 from HYPT. The 42 healthy participants, who cover the age range between 23 and 75 years, form the subjects of the control groups of the four studies with systemic vascular pathologies included in this investigation. In these healthy study participants the corresponding disease was not diagnosed.

**TABLE 1:** Overview of the origin of biopsies evaluated in this investigation.

**Original studies**	**Reference(s)**	**Study groups**	**Biopsies (N)**	**Capillaries analyzed by TBIA (N)**
Endurance exercise	Rosler et al., 1985	1. Participants	10	400
		2. Participants after Exercise	10	400
Angiogenesis responders (AR) and non-responders (NR)	Suter et al., 1995	3. AR-Participants	6	240
		4. AR-Participants after Exercise	6	240
		5. NR-Participants	6	240
		6. NR-Participants after Exercise	6	240
Hypertension	Nyberg et al., 2012	7. Normotensives	10	400
		8. Normotensives after Exercise	10	400
		9. Hypertensives	9	360
		10. Hypertensives after Exercise	9	360
Diabetes	Winding et al., 2018	11. Diabetics	10	400
		12. Diabetics after Exercise	10	400
Intermittent claudication (IC)	Hoier et al., 2013; Walker et al., 2016	13. Participants	10	383
		14. IC patients	14	537

*Shown are the original studies (including their partition into study groups) that were originally performed by our collaborators, as well as number of biopsies and capillary profiles randomly selected for the morphometric analysis presented here. A total of 5,000 capillary profiles from 126 biopsies were assessed. For further information about the study groups, see Supplementary Table 1.*

After local subcutaneous analgesia (20% lidocaine), muscle biopsies were taken from the VL by authorized medical practitioners using Bergstroem needles. Pre- and post-training biopsies were spaced approximately 1 cm apart. Post-training biopsies were collected 24 h after the last exercise bout. Skeletal muscle biopsies were chemically fixed in a 6.25% (v/v) glutaraldehyde solution buffered with 0.1 M sodium cacodylate–HCl (pH 7.4) and stored at 4°C until analysis, as previously described (Baum et al., 2015).

Written informed consent was obtained in each case prior to the study beginning. In all investigations, the criteria and ethical guidelines for treatment of human participants conform to the principles outlined in the Declaration of Helsinki were fulfilled. Each study protocol was approved by the local ethics committee responsible for supervision at the time of study execution, i.e., the Ethics Committee of Faculty of Medicine at the University of Bern, Switzerland; the Ethics Committee of Copenhagen and Frederiksberg Communities, Denmark (H-2-2009-096); the Ethics Committee of the Capital Region of Denmark (H-2-2011-070), and the local Human Research Ethics Committee of the Sunshine Coast, Australia (HREC/09/QRBW/321), as described earlier (Rosler et al., 1985; Suter et al., 1995; Nyberg et al., 2012; Hoier et al., 2013; Walker et al., 2016; Winding et al., 2018).

### Mouse Muscle

C57Bl/6-mice were maintained in a conventional animal facility with a fixed 12 h:12 h light:dark cycle on a commercial pelleted chow diet with free access to tap water. At sacrifice, mice were anesthetized with a ketamine/xylazine (100 mg kg^–1^/5 mg kg^–1^) cocktail *via* intraperitoneal injection. Mice were euthanatized by cutting the carotid arteries, and the soleus muscles were removed to be fixed and stored in 2.5% glutaraldehyde in 0.1 M sodium cacodylate–HCl, pH 7.4 (Merck, Darmstadt, Germany) until use. Experiments were performed in accordance with the approvals published by the Cantonal Committee on Animal Welfare Bern (BE 43/18) and the University of Bern conform to the guidelines from Directive 2010/63/EU of the European Parliament on the protection of animals used for scientific purposes.

### Transmission Electron Microscopy

From each VL biopsy, two randomly selected Epon-embedded blocks were used to prepare ultrathin sections (50–60 nm in thickness) with an Ultracut ultramicrotome (Reichert-Jung, Bensheim, Germany). The sections were floated on 200-mesh parlodion-coated copper grids (Plano, Wetzlar, Germany) and contrasted with 0.5% uranyl acetate and 3% lead citrate as previously described (Baum et al., 2015). The inspection of digital micrographs was performed using a transmission electron microscope (TEM; Morgagni M268; FEI, Brno, Czechia).

### Capillary Morphometry

Between 20 and 25 randomly selected electron micrographs of capillary profiles per ultrathin section/Epon-embedded block were photographed in the TEM at final magnifications between 8.900×–14.000×. Micrographs showing capillary profiles with an aspect ratio (ratio of the smallest to largest diameter) of more than 1.2 were considered too obliquely sectioned and were excluded from morphometric evaluation, as previously recommended (Siperstein et al., 1973; Williamson et al., 1976).

Tablet-based image analysis was performed for capillary morphometry, as previously described (Bigler et al., 2016). On electron micrographs of capillary profiles, lines were drawn with a digital pen using ImageJ (NIH, Bethesda, MD, United States) around the capillary lumen (blood:EC transition), along the abluminal EC surface (EC:BM transition), along the BM:endomysium transition, and around the PC surface to obtain values for areas and circumferences. Absolute arithmetic values for the lumen radius, and the EC and BM thicknesses were calculated using formulae previously derived (Bigler et al., 2016):

(1) R⁢a⁢d⁢i⁢u⁢s⁢(l⁢u⁢m⁢e⁢n)=2*A⁢l⁢u⁢m⁢e⁢n/C⁢l⁢u⁢m⁢e⁢n

(2) Thickness(EC)=2*(AEC:BM-APC)/(CEC:BM+Clumen)

(3) Thickness(BM)=2*(ABM:Endo-AEC:BM-Alumen)/(CBM:Endo+CEC:BM+Clumen)

with A representing area, C representing circumference, EC:BM representing length of the EC:BM-transition, and BM:Endo representing length of the BM:endomysium transition. The PC coverage of capillaries was estimated as ratio of the length of the abluminal EC surface subtended by a PC profile to the total abluminal EC surface (Tilton et al., 1985; Egginton et al., 1996).

After training in the TBIA methodology, two researchers independently measured the length of the perimeter of the lumen/EC transition, the EC nucleus profiles and the intraluminal projections (and the sizes of the corresponding enclosed areas) on the images showing the capillary profiles subjected to this investigation. In this analysis, all 5,000 micrographs of the 14 experimental groups were included to ensure comparability of these structural indices, which sometimes varied considerably when carried out by various researchers. If the readings of the two researchers deviated more than 10%, the measurement was repeated. Using the mean of their determinations, “thickness of the EC layer,” “length of lumen radius,” “EC nucleus volume density,” and “intraluminal EC surface enlargement by projections” were calculated. In contrast, the values for “BM thickness” and “PC coverage” were copied from Baum and Bigler (2016), since it was observed that these structural indices deviate only non-significantly between different researchers using TBIA. We would also like to draw attention to the fact that the values for the lumen radius presented here are twice as high as the values that we had previously determined for the four control groups (Bigler et al., 2016) when we unfortunately applied the formula incorrectly.

To obtain EC nucleus area density, the absolute nucleus area was normalized the total EC area. The intraluminal EC surface enlargement by finger-like projections (= cell processes) of about 100 nm diameter width, without intracellular vesicles appearing either singly or in pairs, was calculated as the sum of the luminal endothelial perimeter plus length of the intraluminal projections, divided by the luminal endothelial perimeter. To obtain a structural index of perivascular interaction, estimates of two researchers were averaged for the proportion of capillaries with appearance of loosened or patchy BM appearance between PC and the abluminal surface of EC to quantify “capillaries with disrupted BM between PC and EC.”

In addition, the proportion of peg-sockets junctions (PSJs) between EC and PC was assessed in accordance to previous reports (Allsopp and Gamble, 1979; Egginton et al., 1996; Bigler et al., 2016). Briefly, the relative number of capillary profiles with structures of interest [i.e., single or multiple intracellular interdigitations, pockets and holes (“sockets”) in the cytoplasm] apparently caused by invading projections (“pegs”) of adjacent cells (Bigler et al., 2016), and the proportion of empty sockets, was analyzed. Pegs were documented as profiles of sectioned cell processes, while sockets of the capillary EC or PC were recognized as pockets and holes in the cytoplasm (either singly or in sequence; empty or filled). Close attention had to be paid to PSJ identification, as cytoplasmic EC sockets resemble in shape and size mitochondria profiles and fused vesicles, which are occasionally lost from resin-embedded sections during sample preparation. However, such structures usually have a distinctive electron translucency, while single cytoplasmic vesicles are significantly smaller, so it is considered unlikely these objects could be confused with EC sockets.

For peg-socket junction morphometry, two researchers independently counted the number of EC sockets, empty EC sockets, PC sockets and empty PC sockets on all 5,000 electron micrographs showing the capillary profiles from the 14 study groups. The mean of their counts were taken to calculate the PSJ-related structural indices. If their readings differed by more than 10%, the quantification was repeated together. The absolute values for the PSJ-specific structural indices in the four control groups of non-diseased persons as previously published (Bigler et al., 2016) were neglected here to ensure comparability between the 14 study groups analyzed for the present investigation.

### Serial Block-Face Scanning Electron Microscopy (SBFSEM)

Mouse muscles were processed for SBFSEM according to a specific protocol previously described (Odriozola et al., 2017). Tissue blocks of 1 mm side length were rinsed four-times with 0.15 M sodium cacodylate–HCl, pH 7.4. After final washing with 0.5% Triton X-100 in 0.15 M sodium cacodylate–HCl, pH 7.4, the muscle blocks were stained in 2% (w/v) OsO_4_ (Electron Microscopy Sciences, Hatfield, PA, United States) and 3% potassium ferrocyanide (Sigma-Aldrich, Buchs, Switzerland) in 0.15 M sodium cacodylate–HCl, pH 7.4. The tissue was then rinsed with bi-distilled water to be incubated in pyrogallol in 0.15 M sodium cacodylate–HCl, pH 7.4 (Sigma-Aldrich, Buchs, Switzerland) for enhancement of the staining. Subsequently, tissue blocks were successively incubated in 2% (w/v) OsO_4_, 1% (w/v) uranyl acetate (Sigma-Aldrich, Buchs, Switzerland) and Walton’s lead aspartate (Sigma-Aldrich, Buchs, Switzerland). Between each of these steps, the tissue blocks were rinsed with bi-distilled water and dehydrated by incubation in an ascending ethanol dilution series (20, 50, 70, 90, and twice 100%). The tissue was infiltrated with Durcupan (epoxy resin, Sigma-Aldrich, Buchs, Switzerland) with decreasing concentrations of ethanol (1:3, 1:1, 3:1, undiluted Durcupan). The resin was polymerized for 48 h at 60°C.

Three-dimensional (3D) micrographs of muscle fine structure were produced on a Quanta FEG 250 (FEI, Eindhoven, Netherlands) SBFSEM equipped with a 3View2XP *in situ* ultramicrotome (Gatan, Munich, Germany). Section thickness was set to 50 nm. Micrographs were acquired with a magnification and image dimension resulting in a pixel size at specimen level of 5.5 nm (x/y). Micrographs were acquired in high vacuum mode (1–4 mPa). Acceleration voltage and pixel dwell time was set to 3 KV and 1 μs, respectively. Image acquisition was done with the built-in Gatan back scattered electron detector and image stack post-processing (alignment, normalization) in Gatan’s Digital Micrograph Program. Each field of view consisted of 2048 * 2048 pixels from 70 sections resulting in a volume of 11.0 * 11.0 * 3.5 μm. Final image montage was done in ImageJ.

### Statistics

Numerical data were expressed as mean ± standard deviation. All morphometric data sets were tested by Kolmogorov–Smirnoff with Lillefors correction and Shapiro–Wilk for their normality of distribution prior to statistical analysis. Comparisons among the five studies were assessed using either a Student’s *t*-test (endurance exercise and T2DM studies consisting each of pre- and post-training biopsies as well as IC patients and control biopsies) or a two-way ANOVA (angiogenesis responders/non-angiogenesis responders and normotensives/hypertensives consisting each of pre- and post-training values). One-way ANOVA was used to compare the structural indices in a cross-study manner between each of the untrained patient groups and the pool of study participants without disease diagnosis of the Endurance exercise, AR and NR, Hypertension, Intermittent claudication studies. If the outcome of the ANOVA was significant, *post hoc* Tukey’s tests for multiple pairwise comparisons were implemented. Any relationship with the age of all study participants (pool of the groups with subjects without disease diagnosis of the Endurance exercise, AR and NR, Hypertension, Intermittent claudication studies) was assessed using Spearman’s rank correlation. Statistical significance was calculated as: ANOVA alpha < 0.05; Student’s *t*-test and Tukey’s multiple comparison test alpha < 0.05, 0.01, and 0.001.

## Results

### Compartmental Structure of Skeletal Muscle Capillaries in Pathologies

**FIGURE 1:**
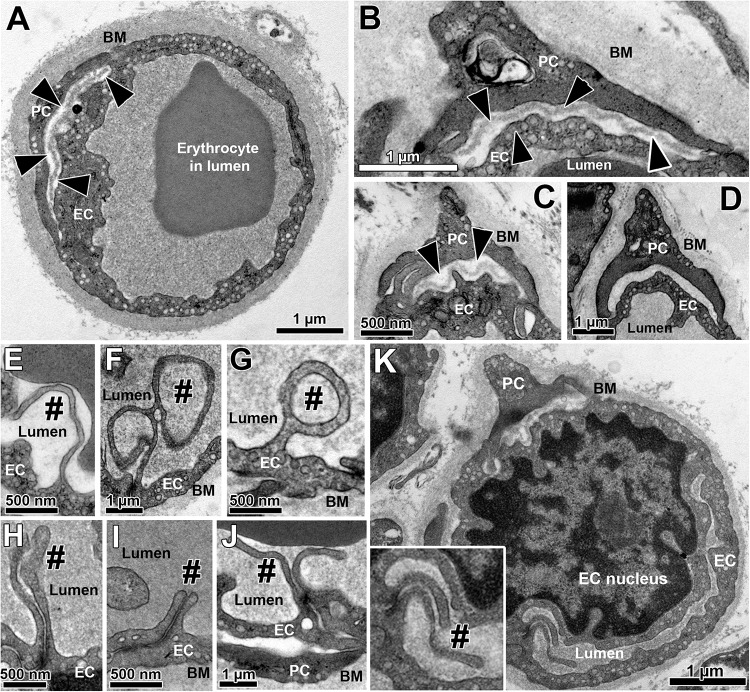
Overview on the fine structure of capillaries in human VL biopsies depicted by transmission electron microscopy. **(A–D)** Capillaries consist of endothelial cell (EC) profiles that form a lumen and are surrounded by a dense basement membrane (BM) in which regularly one or more pericyte (PC) profiles are embedded. The BM area between PC and the abluminal surface of EC (black arrow heads) often appear more disrupted or patchy than at abluminal capillary sites not covered by PC, as shown in three examples **(A–C)**. In other cases **(D)**, this area between EC and PC was completely filled with peri-capillary BM. **(E–K)** Intraluminal projections (cell processes) of variable length and form (#) that branch off from the EC surface into the capillary lumen. The inset in panel **(K)** represents the area of a characteristic pair of intraluminal projections at higher magnification. Note the lariat (loop-like) structure of some intraluminal projections **(F,G)** as well as the distinct EC nucleus of the capillary profile in panel **(K)** that protrudes pronouncedly into the lumen, thereby severely narrowing the area of this compartment. See also Supplementary Figure 2 for colored representation of the capillary structural arrangements shown in this figure, which was added to facilitate the recognition of the cells and compartments.

All skeletal muscle capillary profiles shared a characteristic fine structure, which we quantified by means of morphometry, as presented in Figure 1 and Supplementary Figure 2. The TEM micrographs always showed an evident lumen enclosed by a continuous endothelial cell (EC) layer, and a basement membrane (BM) present at the abluminal surface in which a variable number of pericyte (PC) profiles were embedded. The compartments were distributed differently in the PC: the cytoplasm at the luminal side (including cell processes) usually did not contain subcellular structures, while the abluminal cytoplasm was filled with organelles that appeared frequently fragmented. The area between PC profiles and the EC abluminal surface was often completely packed with BM material (Figure 1D), but could also appear loosened or patchy where the BM layer was clearly disrupted (Figures 1A–C). Usually cell processes protruded from EC into the lumen. These intraluminal projections were seen in the TEM as narrow posts of about 100 nm diameter without intracellular vesicles, appearing either singly or in pairs, which differed considerably in shape and length (Figures 1E–K). When intraluminal projections occurred singly (Figures 1E–G) they often extended either over a long distance into the lumen (>1 μm), where they then appeared to adapt to the shape of erythrocytes (Figure 1E), or form conspicuous lariat (loop-like) structures (Figures 1F,G). In contrast, pairs of intraluminal projections of different lengths that protruded into the lumen were regularly found close to tight junctions between adjacent EC (Figures 1H–K). In some capillary profiles, the EC nucleus was visible and protruded into the lumen, thereby severely narrowing the area of this compartment (Figure 1K).

**FIGURE 2:**
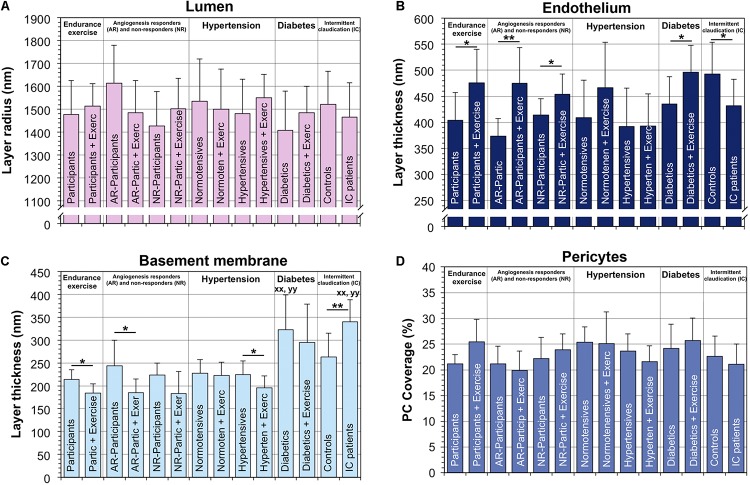
Lumen radius **(A)**, thicknesses of the endothelium **(B)** and the BM layers **(C)** and pericyte coverage **(D)** of capillaries from VL biopsies. The structural indices for the composition of the capillary compartments were determined by TBIA-morphometry. The values for the BM thickness **(C)** and PC coverage **(D)** were previously published in modified form by Baum and Bigler (2016). Data presented as mean ± SD. Statistical significance within studies was tested either with Student’s *t*-test or two-way ANOVA combined with Tukey’s *post hoc* tests, respectively, *P < 0.05; **P < 0.01. Cross-study significance between the collective of healthy participants and the three patient groups was examined by one-way ANOVA combined with Tukey’s *post hoc* tests, ^*xx*^*P* < 0.01 compared to healthy; ^*yy*^*P* < 0.01 compared to HYPT.

The radius of capillary lumen did not significantly differ (*P* ≥ 0.05) between the groups of donors (Figure 2). The EC layer was thicker (*P* < 0.05) in capillary profiles of the VL biopsies taken after training than in those taken before training in four groups (17.3% in exercise study; 27.2% in AR study; 9.2% in NR study, 13.5% in diabetes study). Peri-capillary BM thickness (CBMT) was 38.5 and 45.5% greater (*P* < 0.05) in the T2DM and IC/PAD patient groups, respectively, than in the control group. Lower (*P* < 0.05) CBMT values were observed in the post- versus pre-training biopsies in three groups (−13.7% in exercise study; −24.1% in AR study; −13.1% in HYPT patients). PC coverage of capillaries in VL biopsies was variable among study groups, but not in a consistent manner (*P* ≥ 0.05).

Quantification of minor indices characteristic of capillary fine structure (Table 2) revealed the EC nucleus area density to be similar among groups (*P* ≥ 0.05). The intraluminal EC surface enlargement did not differ between study groups, with the exception of the untrained T2DM group, which exhibited 17.7–39.6% shorter (*P* < 0.05) projections than the other groups. The structural index that was most variable between groups was the proportion of capillaries with a disrupted BM layer between PC and EC, which was higher (*P* < 0.05) in HYPT (33.2%) and T2DM (38.7%) patients than in the controls and IC/PAD patients (20.4 and 25.9%, respectively). Strikingly, exercise training increased the frequency of BM disruption (30.9%, *P* < 0.05) only in the diabetes study.

**TABLE 2:** Characteristic structural indices for the morphology of capillaries in VL biopsies derived from the participants of the 14 study groups.

**Study designation**	**Study groups**	**EC nucleus area density (% of EC area)**	**Intraluminal EC surface enlargement by projections (% of luminal EC surface)**	**Capillaries with disrupted BM between PC and EC (% of capillaries)**
Endurance exercise	1. Participants	16.9 ± 5.1	13.8 ± 3.5	24.1 ± 9.0
	2. Participants after Exercise	16.2 ± 6.7	15.2 ± 1.6	25.6 ± 6.2
Angiogenesis responders (AR) and non-responders (NR)	3. AR-Participants	15.5 ± 5.0	14.3 ± 4.8	25.6 ± 8.3
	4. AR-Participants after Exercise	17.7 ± 6.4	13.0 ± 3.1	24.6 ± 8.1
	5. NR-Participants	18.4 ± 5.7	13.8 ± 3.5	21.5 ± 5.4
	6. NR-Participants after Exercise	16.3 ± 2.1	13.1 ± 2.1	26.0 ± 8.9
Hypertension	7. Normotensives	12.9 ± 5.2	14.0 ± 2.7	25.3 ± 5.4
	8. Normotensives after Exercise	14.5 ± 6.0	16.9 ± 2.4	24.9 ± 9.9
	9. Hypertensives	15.6 ± 6.7	15.8 ± 4.1	33.6 ± 5.8^*,¶^
	10. Hypertensives after Exercise	13.6 ± 4.7	13.7 ± 3.6	37.0 ± 7.3*
Diabetes	11. Diabetics	19.2 ± 5.2	10.2 ± 3.1^¶¶,§^	34.9 ± 8.5^¶¶,§^
	12. Diabetics after Exercise	17.0 ± 5.4	13.7 ± 1.9*	45.7 ± 9.9*
Intermittent claudication (IC)	13. Participants	12.5 ± 6.4	13.6 ± 2.7	28.5 ± 9.8
	14. IC patients	14.2 ± 6.2	12.4 ± 2.6	27.9 ± 6.6

Transmission electron micrographs of capillaries were evaluated by TBIA for assessment of the PC coverage of capillaries, EC nucleus area density and the intraluminal EC surface enlargement by projections, while capillaries with disrupted BM in the area between PC and EC represents the relative frequency. EC, endothelial cell; PC, pericyte. Means ± SD are represented. Statistical significance within studies was tested either with Student’s *t*-test or two-way ANOVA combined with Tukey’s *post hoc* tests, respectively, *P < 0.05 compared to the specific controls. Statistical cross-study significance between the collective of healthy participants and the three patient groups was examined by one-way ANOVA combined with Tukey’s *post hoc* tests, ^¶^*P* < 0.05, ^¶¶^*P* < 0.01 compared to healthy; ^§^*P* < 0.05 compared to HYPT.

Spearman’s rank correlation analyses on the VL biopsies of the 42 participants without disease diagnosis revealed the EC thickness and the BM thickness to be increased (*P* < 0.05) over age (Supplementary Table 2).

### Peg-Socket Junctions Between PC and EC in Skeletal Muscle Capillaries

**FIGURE 3:**
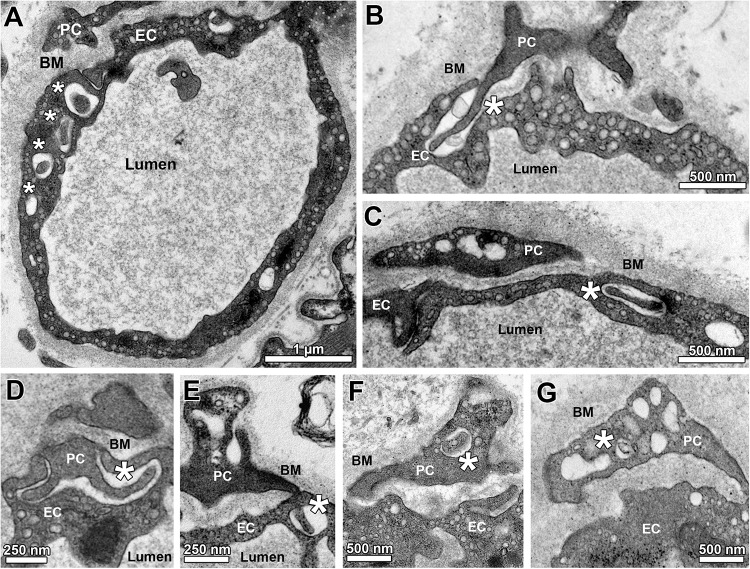
Sub-compartmental structures of capillary profiles (peg-socket junctions). **(A)** Overview on a capillary profile with a sequence of apparent holes (asterisks) in the endothelial layer, which together belong to a defined EC socket. Note that the first hole within this series is filled with a cellular projection that appears to come from the overlying PC profile. **(B)** A PC projection (“peg”) entering the abluminal surface of an EC visualized as a pocket (“socket”) in the EC cytoplasm. **(C)** A hole in the EC profile (“socket”) is filled with a cellular projection presumably arising from the adjacent PC profile. **(D,E)** Additional examples of PC projections (“PC pegs”) that presumably entered the capillary endothelium from its abluminal side to form pockets and (after turning) holes in the EC (“EC sockets”). **(F,G)** Examples for empty and filled holes in PC profiles (“PC sockets”). Asterisks identify sockets in EC **(A–E)** or PC **(F,G)**.

The TEM micrographs were also used to describe and morphometrically assess (Figure 3) the sub-compartmental organization of capillaries in the 14 groups of this investigation, especially the junctional relationship between EC and PC. As illustrated in Figures 3A–E, holes (either singly or as a sequence) were regularly seen in the EC cytoplasm, which likely represent sectioned invaginations (“sockets”). These EC sockets were either empty or filled with profiles of PC cell projections (“pegs”), which traversed the BM to invade the EC abluminal surface, typically in apposition with the overlying PC profile. Most of the EC sockets were clearly less electron-dense than the CBM layer (Figures 3B–E). Some PC profiles contained similar sockets, which likewise appeared either empty or filled with cell fragments (Figures 3F,G).

**FIGURE 4:**
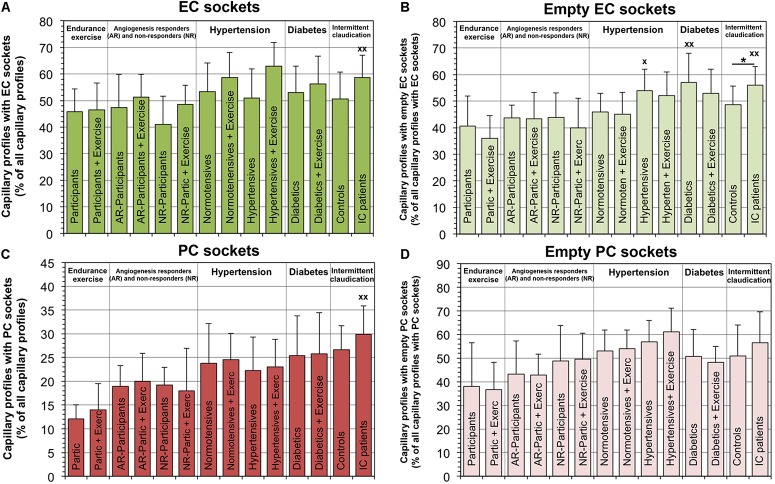
Frequency of peg-socket junctions in EC and PC of capillaries in VL biopsies compared among investigation groups. In each study group, the number of capillary profiles with sectioned sockets (seen as interdigitations or apparent holes in cytoplasm) in EC **(A)** or PC **(C)** as a proportion of total capillary profiles. In addition, the frequency of capillaries with empty sectioned sockets was quantified in EC **(B)** or PC **(D)**. It should be noted that a group comparison of the peg-socket junction indices in the capillaries was previously published with data collected by another researcher using the electron micrographs of the four control groups (Bigler et al., 2016). Data presented as mean ± SD. Significance within studies was tested either with Student’s *t*-test or two-way ANOVA combined with Tukey’s *post hoc* tests, respectively, *P < 0.05. Cross-study significance between the collective of healthy participants and the three patient groups was examined by one-way ANOVA combined with Tukey’s *post hoc* tests, ^*x*^*P* < 0.05, ^*xx*^*P* < 0.01 compared to healthy.

The relative number of capillaries with EC sockets was 21.3% higher (*P* < 0.05) in IC/PAD patients compared to the group of healthy individuals (Figure 4). Interestingly, the abundance of empty EC sockets (without cell profile content) was higher (*P* < 0.05) in the patient groups (20.6% in HYPT, 27.1% in T2DM, 30.0% in IC/PAD) than in healthy participants. The frequency of capillary profiles with PC sockets was 46.6% higher (*P* < 0.05) in IC/PAD patients compared to healthy controls. The incidence of empty PC sockets did not differ (*P* ≥ 0.05) between study groups. Exercise training had only non-significant effects on the PSJ organization between EC and PC. Spearman’s rank correlation analyses on the VL biopsies of the 42 participants without disease diagnosis revealed the frequency of PC sockets, frequency of empty EC sockets and frequency of empty PC sockets to be increased (*P* < 0.05) over age (Supplementary Table 2).

**FIGURE 5:**
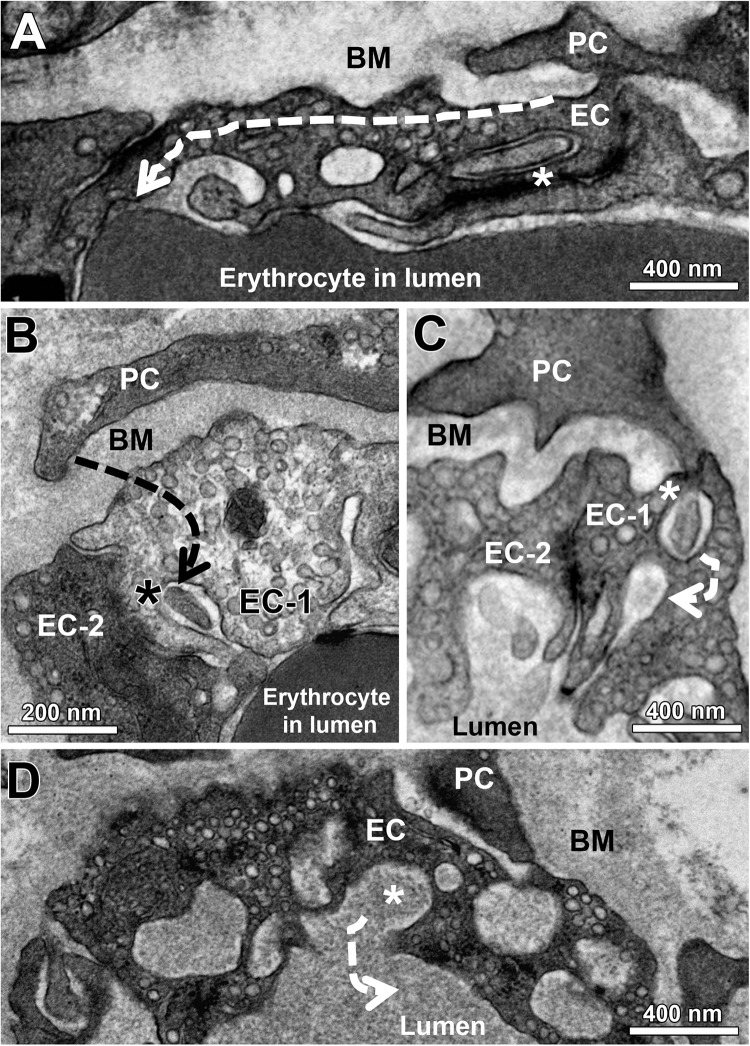
Indirect evidence that EC sockets may merge into the capillary lumen. At the end of a sequence of EC sockets, the last socket section merges into the capillary lumen **(A)**. Note that the first socket section contains a cell projection (asterisk) that appears to come from the overlying PC profile. Facing a PC profile, a socket in EC-1 (asterisk) is clearly connected to the capillary lumen, in which there is a cell fragment originating presumably from the PC. EC-1 and EC-2 are contrasted differently for unknown reason **(B)**. A PC projection **(C)** continues in a sectioned socket of EC-1 (asterisk). In the immediate vicinity of this EC socket is a second (empty) socket, which then opens into the capillary lumen. A sectioned cytoplasmic socket section (asterisk) within a cluster of holes belonging to the same EC socket fuses with the capillary lumen **(D)**. The dashed arrows **(A–D)** indicate the hypothetical course of the EC sockets. See also Supplementary Figure 1 for other examples of EC sockets that merge into the capillary lumen.

**FIGURE 6:**
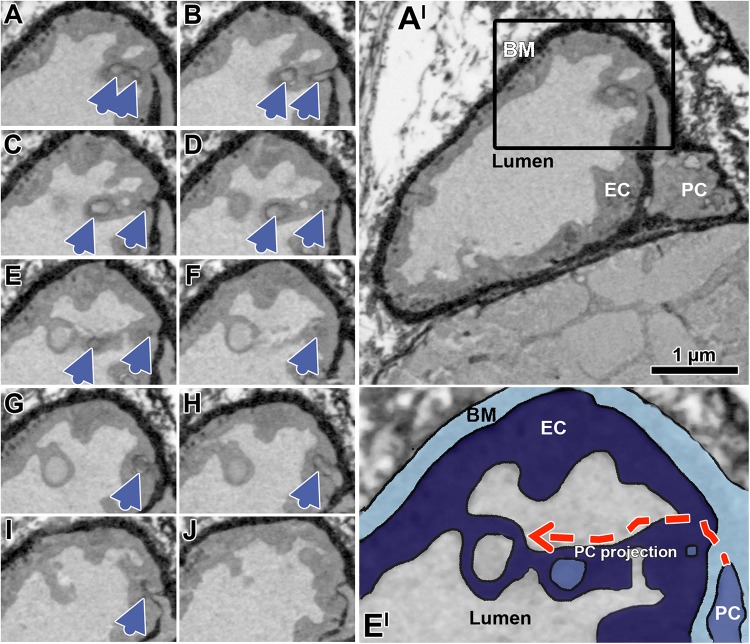
Successive transverse sections of mouse soleus muscle produced by serial block-face scanning electron microscopy (SBFSEM). The sequence of serial sections **(A–J)** taken in 50 nm distance documents a peg projection branches from a PC body to traverse the EC layer, and then to approximate a loop open to the capillary lumen (blue arrow heads). Overview of the overall profile of the capillary **(A’)**, which is partially shown in the black box **(A–J)**. Enlargement **(E’)** of the capillary detail shown in panel **(E)**, colored to facilitate identification of the capillary compartment. The dashed red arrows indicate the hypothetical course of the EC socket. Note that the appearance of the compartments differs from representation in the human VL biopsies previously shown due differences in sample preparation (e.g., contrasting) required for SBFSEM, but while this affects quality of image it has no influence on the interpretation of structure described. A video sequence compiled from the serial sections **(A–J)** as well as additional 70 sections (covering 3.5 μm of the capillary length), which provides an impression of the spatial peg-socket junction structure, can be viewed in Supplementary Material.

When examining the EM micrographs of the capillary profiles, empty sockets were occasionally seen in EC profiles which appeared to be in close proximity to the capillary lumen (Figure 5 and Supplementary Figure 1), an observation not previously reported. To characterize the three-dimensional organization of such structural features, serial sections of skeletal muscle capillaries derived from mice were produced by SBFSEM. This analysis demonstrated that PC pegs traversing the EC might approach EC looped intraluminal projections that were open to the lumen (Figure 6).

## Discussion

In this investigation we characterized the fine structure of capillaries in VL biopsies from individuals with different pathologies and training status by means of TBIA morphometry. We were particularly interested in differences in capillary phenotype of HYPT, T2DM and IC/PAD patients compared to healthy individuals, which may identify structural microangiopathies specific for these systemic vascular pathologies. The major observations were: (1) The lumen radius and extent of PC coverage of capillaries were rather similar in all VL biopsies. In contrast, EC thickness was influenced by the training status, and the CBMT was dependent on both health condition and training status. (2) The structural index of cellular activation, EC nucleus area density, was stable while the intraluminal EC surface enlargement by projections was lower in the group of untrained T2DM patients than in the other study groups. The proportion of capillaries with disrupted BM between PC and EC varied significantly between the study groups. (3) The frequency of EC sockets was similar in most groups, while the abundance of empty EC sockets was highest in HYPT, T2DM, and IC/PAD patient groups. In contrast, the frequency of capillary profiles with PC sockets increased steadily with age, without a significant influence on the incidence of empty PC sockets. For the first time, we describe the occurrence of EC sockets coming in close proximity with the capillary lumen seen in the TEM.

### Compartmental Structure of Skeletal Muscle Capillaries

The lumen radius of skeletal muscle capillaries was statistically indistinguishable between the study groups of VL biopsy donors, to our knowledge the first quantitation of the ultimate structural limit to microvascular perfusion in human pathologies. However, it has been recently speculated that the capillary lumen is reduced in peripheral tissues of HYPT and T2DM patients as a result of low arteriolar blood flow due to increased stiffness of larger arteries, microvascular rarefaction and PC-mediated myogenic response (Strain and Paldanius, 2018). This discrepancy between data and hypothesis may reflect that in the VL biopsies of HYPT and IC/PAD patients used for morphometric analysis, capillary/fiber ratio was not significantly lower than in controls (Gliemann et al., 2015; Baum et al., 2016), which implies that the expected microvascular rarefaction had not yet occurred either due to disease duration or severity.

The capillary EC layer thickness in VL biopsies did not vary greatly between the three patient groups, but was significantly thicker in four out of six groups that underwent exercise-training intervention. A similar EC thickening of skeletal muscle capillaries was observed in rats after ligation of the common iliac artery in combination with mild electrical stimulation, which was attributed to substantial EC swelling (edema) and activation due to the ischemic insult (Hudlicka et al., 2008). This EC swelling is a consistent observation and therefore a potentially functional consequence of EC stress, e.g., caused by elevated cytokines, as observed in muscle biopsies from extreme marathon runners (Jee and Jin, 2012). Since IC/PAD patients suffer from ischemia, a thickening of the EC layer was to be expected in this study group, potentially exacerbating any impairment of vascular function by limiting diffusive exchange capacity. It is therefore possible that other factors [e.g., up-regulated eicosanoid levels (Thomson et al., 1996)] may have countered the potential activating effect of ischemia to prevent EC swelling in these biopsies.

The CBMT in VL biopsies was significantly greater in T2DM and IC/PAD patients, but not in HYPT patients, than in controls confirming previous observations of ourselves (Baum et al., 2016; Mortensen et al., 2019) and others (Makitie, 1977; Williamson and Kilo, 1983). Interestingly, CBM also becomes thicker during “physiological” aging of humans (Bigler et al., 2016), but is reduced after endurance exercise (Williamson et al., 1996; Baum et al., 2015, Gliemann et al., 2015). Thus, the CBMT is a highly variable feature of capillaries in skeletal muscle, considered to be an integrative result of converging local and systemic information from metabolic, hemodynamic and inflammatory forces (Williamson and Kilo, 1983; Baum and Bigler, 2016). A thickened CBM is postulated to have manifold effects on skeletal muscle functions: it may pose a greater barrier for diffusive exchange, may lower the microvascular compliance, and may impede transcytosis of inflammatory cells (Baum et al., 2016). Considering this variety of potential biological functions, it is currently only possible to speculate about the pathophysiological relevance of the thickened CBM in specific patient groups. It has been recently hypothesized that reduced skeletal muscle performance in early stages of metabolic diseases like T2DM and IC/PAD is mainly caused by microvascular dysfunction (Frisbee et al., 2018). Our findings are consistent with this hypothesis, as it suggests that the metabolic needs of muscle fibers may not be adequately met in T2DM and IC/PAD patients due to a thickened CBM representing a significant barrier (in addition to the EC layer and the endomysium) to the flux of oxygen and other substrates. In contrast, the thinner CBM found in the study groups after endurance exercise training may contribute to a better exchange of nutrients and metabolites, and therefore to an increased performance. However, the hypothesis that the thickness has actually a functional impact on the diffusion capacity in skeletal muscle, still needs to be proven experimentally. Pericyte (PC) coverage of capillaries did not significantly vary between study groups, in agreement with previous findings in HYPT (Hernandez et al., 1999), T2DM (Tilton et al., 1981), and IC/PAD (Makitie, 1977) patients, suggesting that this regulation of microvessel diameter (and hence flow resistance) was unaltered.

Among the characteristic minor structural indices assessed, the EC nucleus area density did not significantly differ between the groups of our morphometry study, indicating that neither pathological condition nor training status are accompanied by higher proliferation rates of capillary EC. While this supports an unbiased sampling of capillary profiles (e.g., without preference for capillaries with striking EC nucleus profiles), it underlines the rarity of individual capillary proliferation or apoptosis required to effect microvascular remodeling. Whether the significantly lower intraluminal EC surface enlargement by projections only found in the T2DM group is of functional relevance remains to be investigated, but this may accompany either EC activation and/or increased sensitivity to circulating signals. The proportion of capillaries with disrupted BM between PC and EC was significantly higher in HYPT and T2DM patients at baseline while, in contrast, the proportion of such capillaries did not vary in IC/PAD patients (all compared to controls). A local increased activity of matrix metalloproteinases (MMP), or induction of MMP expression, in response to the onset of systemic vascular pathologies may counteract thickening of CBM. This may occur generally around the capillary and affect compliance or ease of EC sprouting, or particularly between EC and PC affecting paracrine signaling. This is supported by the observation of increased MMP activity in T2DM patients after exercise training (Scheede-Bergdahl et al., 2014), but not in PAD patients (Baum et al., 2007).

When visualized on two-dimensional transmission electron micrographs of capillaries, lariats consistently appear as thin cytoplasmic EC projections that enclose an area, which is occasionally connected to the capillary lumen. When conceived in three-dimensional space, these projections consist of a thin EC wall surrounding a cavity and, thus, may more closely resemble balloons than lariats. However, we prefer to use the latter term as those who are not used to viewing TEM images may not at first recognize a balloon structure.

### Peg-Socket Junctions Between PC and EC in Skeletal Muscle Capillaries

To our knowledge this is the first study in which the appearance of PSJs between EC and PC was quantified in pathological muscle samples. We found more empty EC sockets in the VL biopsies from the three patient groups (HYPT, T2DM, IC/PAD) than in the groups with trained or untrained healthy participants. This observation indicates that PC cell processes are truncated in capillary EC of patients compared to non-diseased controls, consistent with PC retraction (Tilton et al., 1979), which in turn may result in convoluted abluminal membrane leading to an increased membrane surface area. Given that PSJ enhance the mechanical and probably electrical connection between these two cell types, it is possible that the stabilizing effect of PC on microvascular integrity (Hamilton et al., 2010) is weakened in these pathologies. However, a thickened BM may mechanically compensate for this reduction in PC function.

In the TEM, occasionally empty sockets were noticed in EC profiles that appeared to be in close proximity with the capillary lumen, as also observed by others (Blervaque et al., 2019). These recordings support our so far experimentally unproven hypothesis that (empty) EC sockets cross the entire EC and open apically to the lumen, thus allowing a direct communication between PC and the blood circulation and could directly sense specific parameters (e.g., shear stress, pH, chemical composition).

### Changes in the Fine Structure of Skeletal Muscle Capillaries in Human Pathologies

**FIGURE 7:**
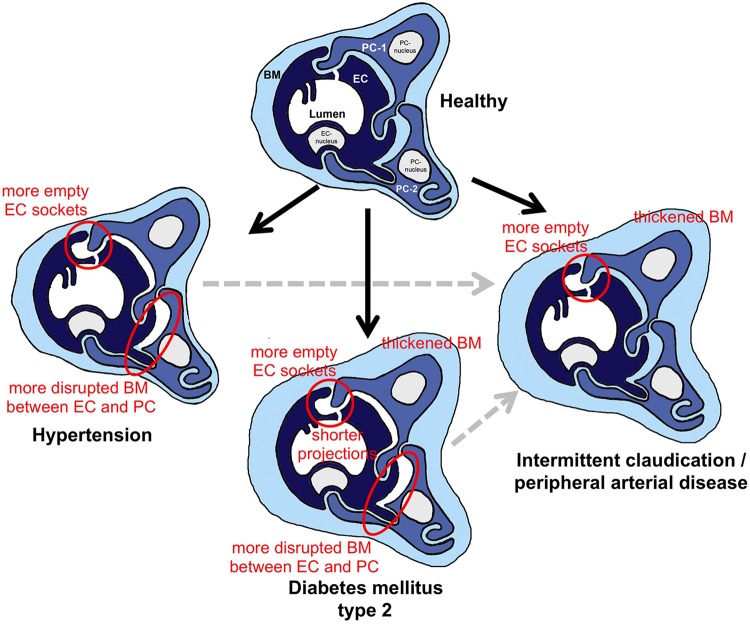
Schematic summary of the morphometric results from this investigation regarding structural changes in skeletal muscle capillaries in HYPT, T2DM or IC/PAD patients. In the three capillary depictions of the patient groups, the structures that were altered in comparison to healthy participants are highlighted. To simplify identification of these changes in morphology, red circles surround important features. When interpreting the data, it should be noted that the HYPT and T2DM, IC/PAD patients were originally healthy (black arrows). In addition, it should be appreciated that IC/PAD may develop from HYPT or T2DM (dashed gray arrows). BM, basement membrane; EC, endothelial cell; PC, pericyte.

The schematic drawing in Figure 7 summarizes the outcome of our morphometric analysis to characterize the phenotype of capillaries in VL biopsies from HYPT, T2DM and IC/PAD patients. In comparison to non-diseased individuals: (1) HYPT patients exhibited more frequent disruption of BM between PC and EC, and more empty EC sockets, (2) T2DM patients had a thicker CBM, shorter intraluminal projections (cell processes), more frequent disruption of BM between PC and EC and more empty EC sockets as well as (3) IC/PAD patients had a thicker CBM and more empty EC sockets.

Although the three chronic systemic vascular pathologies studied are accompanied by different etiologies, they all exert influences on the peripheral microvascular system. HYPT affects the hemodynamics in all sections of the circulation, including the capillaries, long before organ dysfunction becomes clinically manifest (Jung et al., 2013). T2DM primarily modulates the cell metabolism including that of capillary EC (Brownlee, 2001). IC/PAD usually develops as a result of arteriosclerotic stenosis in patients who have often previously suffered from HYPT and/or T2DM, so that the skeletal muscle capillaries are exposed not only to ischemia/hypoxia, but also to altered hemodynamic conditions (e.g., low flow, reduced shear stress) and/or a shifted metabolic milieu (e.g., mitochondrial dysfunction).

Despite the different personnel undertaking morphometry and the differences in absolute values, all structural indices identified in Bigler et al. (2016) as being statistically changed over age (BM thickness, frequency of empty EC sockets, frequency of empty PC sockets) were also found to be significantly changed over age in this study (additional structural indicators significantly changed: EC thickness, frequency of PC sockets). When comparing the values for the five age-dependent structural indices between the patient groups and their control groups, only two indices were significantly changed in the same direction as in the correlation analysis, which means that the age-related effect is reinforced: the CBM thickness was in T2DM and IC/PAD patient groups higher and the frequency of empty EC sockets was higher in the HYPT, T2DM, and IC/PAD patients groups than in the control group. Remarkably, the mean values for these two structural indices in the patient groups were significantly higher than the mean values in each control group of the participants without disease diagnosis. Therefore, we deem it is unlikely that the CBM thickening and increase in the frequency of empty EC sockets observed in the patient groups is due to aging.

Inevitably, some methodological limitations may limit the validity of our suggested significance of these altered structural features. (1) We cannot rule out technical distortion during tissue treatment (e.g., glutaraldehyde fixation shrinkage), although all biopsies were treated in the same way. Note, therefore, that the absolute values for the structural indices shown are not directly considered. (2) The arithmetic values given in this study are only estimates of structural limits to performance. For a more functional interpretation of morphometric findings, such as their possible relationship to oxygen and substrate flux, other indices may be more appropriate, e.g., the harmonic mean barrier thickness (Weibel, 1979), which accounts for the fact that thinner segments contribute proportionally more to diffusion than thicker ones. (3) All studies with patient samples show a bias in the disease duration, as the systemic vascular pathologies analyzed here are usually not diagnosed until the onset of symptoms.

## Conclusion

If one considers the nature of the pathologies when interpreting the findings from our comparative morphometric analysis, then four conclusions may be drawn: (1) Shortening of intraluminal projections appears to be a structural rearrangement of skeletal muscle capillaries prevalent in T2DM. (2) Thickening of CBM is a structural feature common for T2DM and PAD, which may contribute to changes in oxygen tension and/or substrate metabolism reported in these systemic vascular pathologies. (3) Shortening of EC-entering PC projections (deduced from the observation of increased frequency of empty EC sockets) is a phenomenon commonly observed in each of the three pathologies, and thus may contribute to a disturbed EC-PC signaling in skeletal muscle capillaries.

The term “microangiopathy” is not consistently used in the scientific literature, but describes (in part very different) functional and structural changes of the microcirculation, e.g., diabetic retinopathy is associated with EC proliferation, while diabetic nephropathy involves apoptotic degeneration of EC. It may therefore be more appropriate and helpful to discriminate between functional and structural microangiopathies. With this distinction it would be possible to compare the morphological changes in the microcirculation of patients with various chronic systemic vascular pathologies, not only in skeletal muscle but also in other tissues. Furthermore, defining structural microangiopathic features could also be relevant for the functional validation of *in vitro* studies recently undertaken to re-form the interaction of blood vessels within organoids (Wimmer et al., 2019). Thus, the outcome of this investigation may contribute to the current understanding of the etiology and progression of chronic systemic vascular pathologies at various levels.

## Data Availability Statement

The datasets generated for this study are available on request to the corresponding author.

## Ethics Statement

The studies involving human participants were reviewed and approved by the Ethics Committee of Faculty of Medicine at the University of Bern, Switzerland, the Ethics Committee of Copenhagen and Frederiksberg Communities, Denmark, the Ethics Committee of the Capital Region of Denmark, and Human Research Ethics Committee of the Sunshine Coast, Australia. The patients/participants provided their written informed consent to participate in this study. The animal study was reviewed and approved by Cantonal Committee on Animal Welfare Bern.

## Author Contributions

OB, BZ, ST, AZ, SE, and JaB contributed to the conception and design of the study. JoB, SB, and AO performed the experiments. OB and ST performed the statistical analysis. OB wrote the first draft of the manuscript. BZ, AZ, SE, and JaB wrote sections of the manuscript. All authors contributed to manuscript revision, and read and approved the submitted version.

## Conflict of Interest

The authors declare that the research was conducted in the absence of any commercial or financial relationships that could be construed as a potential conflict of interest.
