# Supplementary material for: Structural Microangiopathies in Skeletal Muscle Related to Systemic Vascular Pathologies in Humans
Source: Front Physiol. 2020 Feb 5;11:28. doi: 10.3389/fphys.2020.00028 (PMC7013089; doi:10.3389/fphys.2020.00028)
Supplement: Supplementary file 2 [file Data_Sheet_1.PDF]

Supplementary Table 1: Overview of the studies and the references in which the findings of the capillary morphometry obtained by grid morphometry were originally published.

Study designation	Study groups	Study design	Reference
Endurance exercise	1. Participants<br>2. Participants after Exercise	10 male Participants (30.5 ± 5.0 y old; VO_2max: 51.6 ml*min^{-1}*kg^{-1}) undergoing bike training up to 95% HRmax 5x per wk for 30 min for 8 wks	Baum et al., 2015
Angiogenesis responders AR and non-responders NR)	3. AR-Participants<br>4. AR-Participants after Exercise<br>5. NR-Participants<br>6. NR-Participants after Exercise	12 male Participants (36.3 ± 6.3 y old, VO_2max: 38.3 ml*min^{-1}*kg^{-1}) undergoing jogging training up to 90% HRmax<br>4x per wk for 30 min for 6 mo	Baum et al., 2015
Hypertension	7. Normotensives<br>8. Normotensives after Exercise<br>9. Hypertensives<br>10. Hypertensives after Exercise	9 Normotensives (47.4 ± 1.4 y old, VO_2max: 34.4 ml*min^{-1}*kg^{-1})<br>10 Hypertensives (46.1 ± 1.2 y old, VO_2max: 33.4 ml*min^{-1}*kg^{-1}) undergoing ergometer HIT up to 95% HRmax<br>2-3x per wk for 30 min for 8 wks	Gliemann et al., 2015
Diabetes	11. Diabetics<br>12. Diabetics after Exercise	10 Diabetics (55.8 ± 8.3 y old; BMI: 27.0 kg*m^{-2}) undergoing ergometer training up to 95% HRmax<br>2-3x per wk for 12 wks	Mortensen et al., 2018
Intermittent claudication (IC)	13. Participants<br>14. IC/PAD patients	10 Participants<br>(64.0 ± 8.7 y old, ABI: 1.20)<br>14 Intermittent claudication patients<br>(68.1 ± 4.5 y old, ABI: 0.73)	Baum et al., 2016

ABI, ankle-brachial index; BMI, body mass index; HR_{max}, maximum heart rate; $VO2_{max}$, maximal oxygen consumption; PAD, peripheral arterial disease

Baum, O., Gubeli, J., Frese, S., Torchetti, E., Malik, C., Odriozola, A., Graber, F., Hoppeler, H., and Tschanz, S.A. (2015). Angiogenesis-related ultrastructural changes to capillaries in human skeletal muscle in response to endurance exercise. J. Appl. Physiol. (1985) 119, 1118-1126. doi: 10.1152/japplphysiol.00594.2015

Baum, O., Torchetti, E., Malik, C., Hoier, B., Walker, M., Walker, P.J., Odriozola, A., Graber, F., Tschanz, S.A., Bangsbo, J., Hoppeler, H., Askew, C.D., and Hellsten, Y. (2016). Capillary ultrastructure and mitochondrial volume density in skeletal muscle in relation to reduced exercise capacity of patients with intermittent claudication. Am. J. Physiol. Regul. Integr. Comp. Physiol. 310, R943-951. doi: 10.1152/ajpregu.00480.2015

Gliemann, L., Buess, R., Nyberg, M., Hoppeler, H., Odriozola, A., Thaning, P., Hellsten, Y., Baum, O., and Mortensen, S.P. (2015). Capillary growth, ultrastructure remodelling and exercise training in skeletal muscle of essential hypertensive patients. Acta Physiol. (Oxf) 214, 210-220. doi: 10.1111/apha.12501

Mortensen, S.P., Winding, K.M., Iepsen, U.W., Munch, G.W., Marcussen, N., Hellsten, Y., Pedersen, B.K., and Baum, O. (2018). The effect of two exercise modalities on skeletal muscle capillary ultrastructure in individuals with type 2 diabetes. Scand. J. Med. Sci. Sports. doi: 10.1111/sms.13348
