# Supplementary material for: Structural Microangiopathies in Skeletal Muscle Related to Systemic Vascular Pathologies in Humans
Source: Front Physiol. 2020 Feb 5;11:28. doi: 10.3389/fphys.2020.00028 (PMC7013089; doi:10.3389/fphys.2020.00028)
Supplement: Supplementary file 3 [file Data_Sheet_2.PDF]

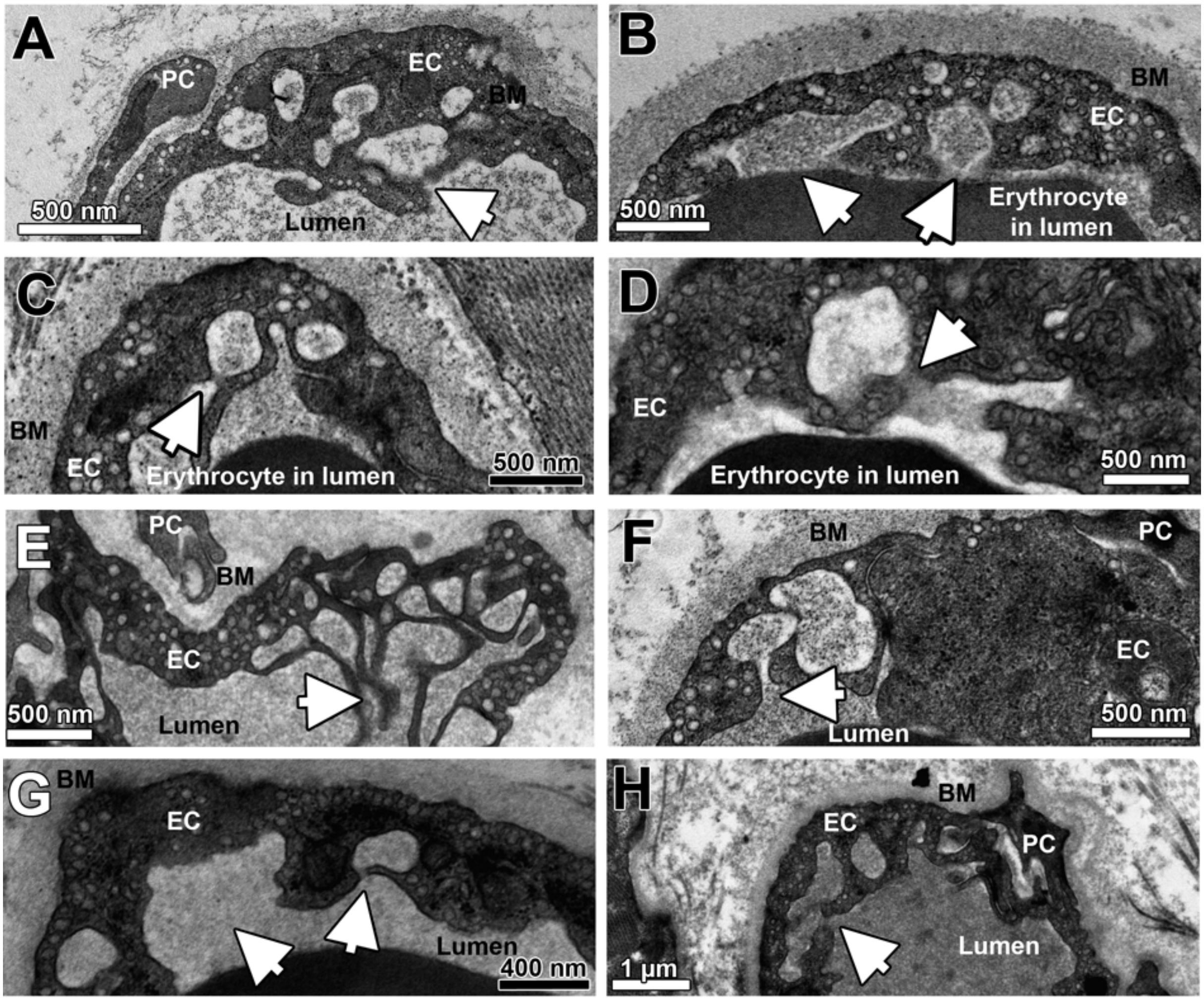

Supplementary Figure 1: Further examples of EC sockets merging into the capillary lumen. The white arrows indicate the presumed opening into the lumen.
