# Supplementary material for: Structural Microangiopathies in Skeletal Muscle Related to Systemic Vascular Pathologies in Humans
Source: Front Physiol. 2020 Feb 5;11:28. doi: 10.3389/fphys.2020.00028 (PMC7013089; doi:10.3389/fphys.2020.00028)
Supplement: Supplementary file 4 [file Data_Sheet_3.PDF]

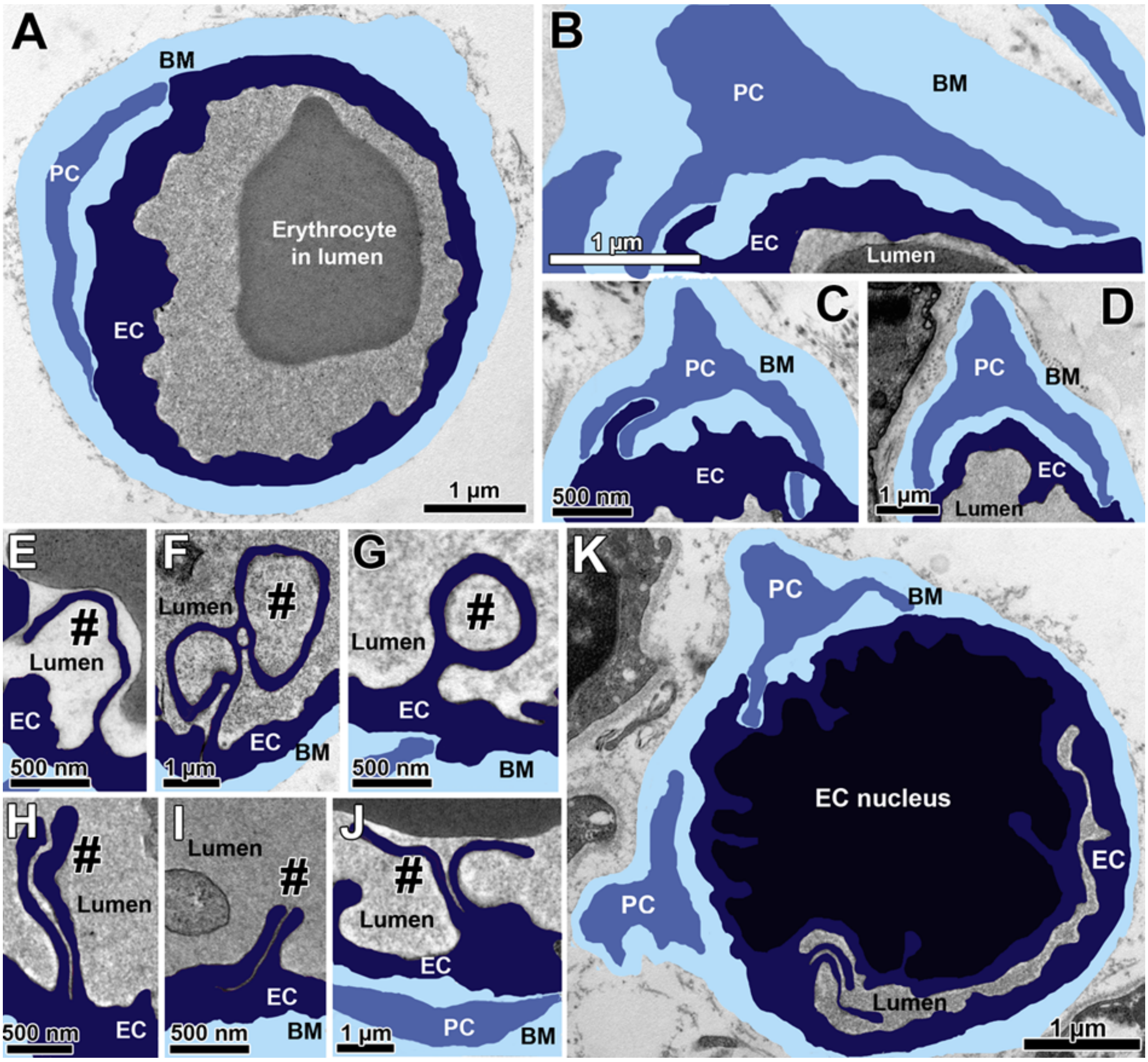

Supplementary Figure 2: Colored representation of the structural arrangement of capillaries in human skeletal muscle. In order to facilitate the recognition of the capillary fine structures, cells and compartments of the micrographs shown in Figure 1 were marked with different colors as indicated.
