# Supplementary material for: Structural Microangiopathies in Skeletal Muscle Related to Systemic Vascular Pathologies in Humans
Source: Front Physiol. 2020 Feb 5;11:28. doi: 10.3389/fphys.2020.00028 (PMC7013089; doi:10.3389/fphys.2020.00028)
Supplement: Supplementary file 5 [file Data_Sheet_4.PDF]

Supplementary Table 2: Spearman's rank correlation analysis to assess the relationship between the age of the healthy participants and the structural indices of capillaries in their VL muscle biopsies.

**Index**	**Correlation**	**P**	**Significance level**
Lumen radius	0.14	0.39	
EC thickness	0.43	0.003	**
BM thickness	0.45	0.002	**
PC coverage	0.25	0.12	
EC nucleus area density	-0.29	0.07	
Intraluminal EC surface enlargement	0.02	0.89	
Frequency of capillaries with disrupted BM between PC and EC	0.15	0.36	
Frequency of capillaries with EC sockets	0.16	0.29	
Frequency of capillaries with empty EC sockets	0.30	0.04	*
Frequency of capillaries with PC sockets	0.55	0.0002	***
Frequency of capillaries with empty PC sockets	0.31	0.04	*

The 42 healthy participants, who cover the age range between 23 and 75 years, form the subjects of the control groups of the four studies with systemic vascular pathologies included in this investigation. In the healthy study participants the corresponding disease was not diagnosed. For definition of indices and their units, see manuscript. Statistical significance: *$P < 0.05$; **$P < 0.01$; ***$P < 0.001$
